# GD3 Synthase Overexpression Sensitizes Hepatocarcinoma Cells to Hypoxia and Reduces Tumor Growth by Suppressing the cSrc/NF-κB Survival Pathway

**DOI:** 10.1371/journal.pone.0008059

**Published:** 2009-11-26

**Authors:** Josep M. Lluis, Laura Llacuna, Claudia von Montfort, Cristina Bárcena, Carlos Enrich, Albert Morales, José C. Fernandez-Checa

**Affiliations:** 1 Department of Cell Death and Proliferation, IIBB-CSIC, and Liver Unit, Hospital Clinic, IDIBAPS-CIBEK, CIBEREHD, Barcelona, Spain; 2 Departament de Biologia Cellular, Facultat de Medicina, Universitat de Barcelona, Barcelona, Spain; 3 Research Center for Alcoholic Liver and Pancreatic Diseases, Keck School of Medicine of the University of Southern California, Los Angeles, California, United States of America; University of Hong Kong, Hong Kong

## Abstract

**Background:**

Hypoxia-mediated HIF-1α stabilization and NF-κB activation play a key role in carcinogenesis by fostering cancer cell survival, angiogenesis and tumor invasion. Gangliosides are integral components of biological membranes with an increasingly recognized role as signaling intermediates. In particular, ganglioside GD3 has been characterized as a proapoptotic lipid effector by promoting cell death signaling and suppression of survival pathways. Thus, our aim was to analyze the role of GD3 in hypoxia susceptibility of hepatocarcinoma cells and *in vivo* tumor growth.

**Methodology/Principal Findings:**

We generated and characterized a human hepatocarcinoma cell line stably expressing GD3 synthase (Hep3B-GD3), which catalyzes the synthesis of GD3 from GM3. Despite increased GD3 levels (2–3 fold), no significant changes in cell morphology or growth were observed in Hep3B-GD3 cells compared to wild type Hep3B cells under normoxia. However, exposure of Hep3B-GD3 cells to hypoxia (2% O_2_) enhanced reactive oxygen species (ROS) generation, resulting in decreased cell survival, with similar findings observed in Hep3B cells exposed to increasing doses of exogenous GD3. In addition, hypoxia-induced c-Src phosphorylation at tyrosine residues, NF-κB activation and subsequent expression of Mn-SOD were observed in Hep3B cells but not in Hep3B-GD3 cells. Moreover, MnTBAP, an antioxidant with predominant SOD mimetic activity, reduced ROS generation, protecting Hep3B-GD3 cells from hypoxia-induced death. Finally, lower tumor growth, higher cell death and reduced Mn-SOD expression were observed in Hep3B-GD3 compared to Hep3B tumor xenografts.

**Conclusion:**

These findings underscore a role for GD3 in hypoxia susceptibility by disabling the c-Src/NF-κB survival pathway resulting in lower Mn-SOD expression, which may be of relevance in hepatocellular carcinoma therapy.

## Introduction

Hypoxia is a prominent characteristic of advanced solid tumors and a major determinant of malignant progression and therapy responsiveness [1]–[2]. Although, the molecular mechanisms responsible for the cellular adaptation to hypoxia is still under debate, with prolylhydroxylases playing a fundamental role [3], mitochondrial reactive oxygen species (ROS) generation is believed to contribute to this process, as hypoxia-induced mitochondrial ROS has been shown to determine HIF-1α stabilization and NF-κB activation [4]–[5].

Despite a key structural role in biological membranes, glycosphingolipids (GSLs) are increasingly recognized as secondary intermediates that participate in various cellular processes, including cell adhesion, differentiation, signal transduction and cell death [6], [7]. In particular, ganglioside GD3 (GD3) has been identified as a lipid death effector [8], with a dual mechanism involving its interaction with mitochondria leading to activation of apoptotic pathways [9], [10] and the suppression of survival programs mediated by NF-κB activation [11], [12]. In addition, the acetylation of GD3 by O-acetyl disialoganglioside synthase antagonizes its apoptotic potential and has been shown to regulate tumor cell growth and differentiation [13], [14]. Thus, GD3 acetylation represents a novel mechanism whereby specific tumor cells with elevated GD3 levels escape from GD3-induced cell death [15].

We have recently shown that mitochondrial ROS play a dual role in hypoxia signaling [5]. While hypoxia-induced ROS protected cancer cells by NF-κB activation through a c-Src-dependent mechanism, ROS overproduction following mitochondrial GSH depletion sensitized cancer cells to hypoxia. Given the role of exogenous GD3 in chemotherapy susceptibility [16] and its ability to disable survival pathways dependent on NF-κB activation [11], [12], the purpose of the present study was to examine the role of GD3 in hypoxia susceptibility and *in vivo* tumor growth. To this aim, we generated and characterized a human hepatocarcinoma cell line stably transfected with GD3 synthase. Our results indicate that overexpression of GD3 synthase increases the levels of GD3, which is synthesized from endogenous GM3, rendering Hep3B cells susceptible to hypoxia-induced ROS generation by suppressing the hypoxia-mediated NF-κB activation via c-Src, which results in lower expression of the κB-dependent antioxidant Mn-SOD. Moreover, GD3 synthase overexpression reduces tumor growth *in vivo* in Hep3B-GD3 xenografts. Thus, these findings identify GD3 as a potential relevant therapeutic agent to switch hypoxia from a cancer-promoting to a cancer-threatening environment.

## Results

### Stable Expression of GD3 Synthase in Hep3B Cells

**Figure 1:**
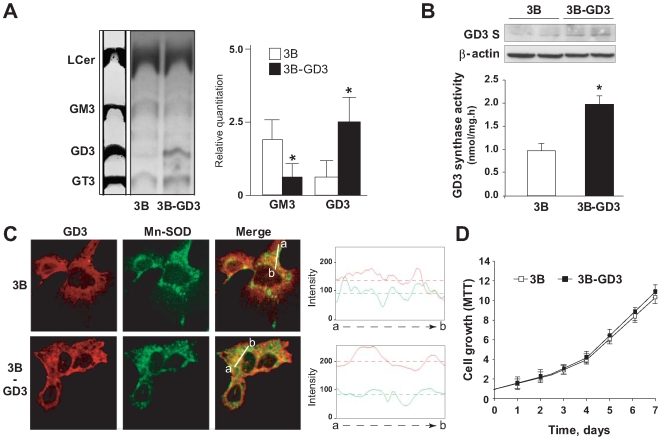
Characterization of GD3 synthase-overexpressing Hep3B cells. *A.* Representative image of lipids extracts resolved by HPTLC from Hep3B cells transfected with and empty vector (3B) or overexpressing GD3 Synthase (3B-GD3) isotopically labeled with ^3^H-galactose, and quantification of three independent experiments. Commercial non-labeled standards were run in parallel, being TLC plates stained with 5% orcinol solution. *p<0.05 vs. Hep3B control cells. *B.* GD3 synthase activity was measured in control Hep3B (3B) and Hep3B GD3-expressing cells (3B-GD3). GD3 Synthase protein levels were analyzed by western blot and compared with β-actin levels, used as a control. *C.* Confocal immunofluorescence was visualized in empty-vector containing and GD3 Synthase overexpressing Hep3B cells using antibodies anti-GD3 and anti-Mn-SOD. The graphs on the right panel represent the fluorescence intensity profile plotted from a to b direction in the merge images for the different cell lines (Mn-SOD in green and GD3 in red). *D.* Cell growth under normoxic conditions (n = 3). *p<0.05 vs. Hep3B control cells.

**Figure 2:**
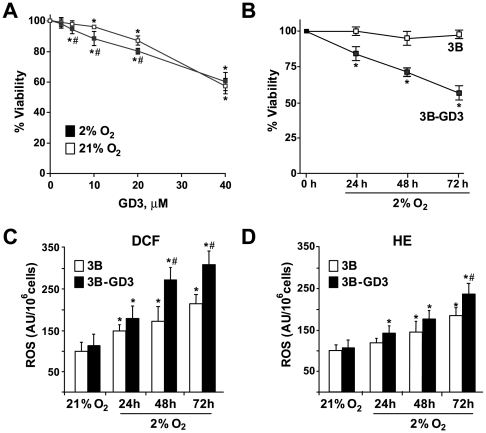
GD3 ganglioside sensitizes Hep3B cells to hypoxia and potentiates ROS generation. *A.* Cell death in Hep3B cells growing under normoxia or hypoxia for 48 h, and treated with increasing doses of GD3. Cell viability measured 24 h later (n = 3). *B.* Time-dependent viability of Hep3B and Hep3B-GD3 cells exposed to 2% O_2_ (n = 3). *C, D.* ROS generation evaluated with fluorescent probes HE (5 µM) and DCF (1 µM) during 30 min at 37°C (n = 4). *p<0.05 vs. Hep3B cells. #p<0.05 vs. hypoxic Hep3B cells.

Many tumors cells exhibit enhanced synthesis of selected gangliosides and abnormalities in GSLs biosynthesis have been implicated in the oncogenesis and malignancy of cancer cells, particularly in neuroectodermal tumors (melanoma and neuroblastoma) [17]. However, the pattern of GSLs expression in human hepatocarcinoma cells has been less explored, with reports showing a low expression of endogenous GD3 in specific cell lines [18], [19]. In fact, while GM3 is the most abundant ganglioside in human liver (around 90%) followed by GD3 (around 5%) [20], in pathological conditions such as cirrhosis or HCC, changes in the ganglioside pattern of GM3 and GD3 and the appearance of minor species (e.g. GM2 or GD1a) are common features [21]. Given the dual role of GD3 in cell death regulation [8]–[11], our aim was to examine the contribution of GD3 to hypoxia susceptibility in human hepatocarcinoma cells following GD3 synthase overexpression. We first characterized the pattern of endogenous GSLs in Hep3B cells, stably transfected with an empty pcDNA vector, following incubation with [^3^H]-galactose and subsequent TLC analysis (**Figure 1A**), and compared it with the levels observed in Hep3B cells transfected with GD3 synthase full-length cDNA (Hep3B-GD3). As expected from the overexpression of GD3 synthase, the predominant changes in the GSLs profile of Hep3B-GD3 cells included a decrease in the content of GM3 accompanied by a significant increase in GD3 levels, as expected after GD3 synthase overexpression, without changes in other glycosphingolipids. Consistent with these changes, we analyzed both the expression of GD3 synthase by western blot and its activity (**Figure 1B**). As seen, Hep3B-GD3 cells exhibited a significant increased in either protein level and activity compared to control Hep3B cells transfected with the empty vector. This enhanced expression in GD3 synthase translated in increased GD3 levels (2–3 fold) and a subsequent decrease in endogenous GM3 content (**Figure 1A**). Moreover, although plasma membrane is a predominant site for GSL, GD3 in particular has been located in other subcellular organelles, including mitochondria in various cell types [10], [22]–[23]. Hence, we next analyzed by confocal immunofluorescence if changes in the cell morphology or in the subcellular distribution of GD3 were induced by the accumulation of GD3 in Hep3B-GD3 cells, as previously measured by HPTLC (**Figure 1C**). As seen, Hep3B-GD3 cells displayed enhanced GD3 levels both at the cell surface and in internal membranes, most likely including mitochondria, as revealed by the merged fluorescence upon mitochondrial staining with antibody anti-Mn-SOD (**Figure 1C**). Despite the increase in GD3 levels, however, the morphology and growth rate of Hep3B-GD3 cells under normoxia did not differ with respect to Hep3B cells (**Figure 1D**), suggesting that the intracellular GD3 levels in Hep3B-GD3 cells were insufficient to cause cell death or ROS generation. Indeed, under these growing conditions we did not detect a significant increase in ROS despite the presence of GD3 in mitochondria (not shown). These findings indicate that stimulated GD3 levels following GD3 synthase overexpression do not exert any obvious effects in Hep3B-GD3 cells grown under normoxia. The lack of endogenous ROS formation in Hep3B-GD3 cells under normoxia is of interest given the potential trafficking of GD3 to mitochondria, especially in light of the reported observations that the targeting of GD3 to mitochondria results in ROS formation [10], [23]. Thus, although we did not examine the threshold for the mitochondrial stimulation of ROS formation by GD3 in Hep3B-GD3 cells, these data imply that the mitochondrial trafficking of GD3 in the transfected cells may be insufficient to cause significant ROS generation, consistent with the lack of effects on cell morphology and growth. In support for this, increasing doses of exogenous GD3 in Hep3B cells grown in normoxia caused cell death (**Figure 2A**) and stimulated ROS generation (not shown), in line with previous observations [9].

### Hep3B-GD3 Cells Become Susceptible to Hypoxia-Induced ROS

Hypoxia has been shown to stimulate ROS production [4], [24] whose impact on cell survival is controlled by NF-κB activation [5]. Since GD3 has been shown to prevent NF-κB activation and suppress κB-dependent gene expression [11], [12], [25], we next analyzed the susceptibility of Hep3B-GD3 cells to hypoxia. Interestingly, in contrast to the outcome observed in normoxia, we observed a time-dependent decrease in the viability of Hep3B-GD3 cells following hypoxia exposure (2% O_2_) compared to control Hep3B cells transfected with empty vector examined by MTT (**Figure 2B**), LDH leakage (not shown) or flow cytometry analysis of propidium iodide exclusion in non permeabilized cells (**Figure S1**). Of note, all selected clones with enhanced GD3 overexpression (over 2 fold increase in activity) displayed similar sensitivity to hypoxic culture with respect to control Hep3B cells (data not shown). Moreover, there was no changes in cell cycle distribution (**Figure S2**), and administration of a pan-caspase inhibitor (qVD-OPH) did not rescue Hep3B-GD3 cells from hypoxia-mediated cell death (**Figure S3**), suggesting that Hep3B-GD3 cells die by necrosis when grown under hypoxia. We next examined the effect of GD3 synthase downregulation in hypoxia susceptibility. RNA interference against GD3 synthase through transfection of specific siRNAs reduced the mRNA and protein levels in Hep3B cells compared to Hep3B cells transfected with scrambled control siRNA (**Figure S4**). However, this outcome did not change the viability of Hep3B cells neither under normoxic nor hypoxic conditions (102.1%±3.8 and 96.6%±5.5 respectively, examined by MTT, compared to Hep3B cells transfected with control siRNA, 97.5%±5.4 and 95.7%±4.9, respectively), although the siRNA against GD3 synthase reversed the susceptibility of Hep3B-GD3 cells to hypoxia. Indeed, the downregulation of GD3 synthase in Hep3B-GD3 cells conferred hypoxia resistance similar to Hep3B cells (viability of 86.6%±6.9 vs. 60.8%±4.5 in Hep3B-GD3 cells after 3 days under 2% O_2_ hypoxia). Coupled with the dose-dependent effect of exogenous GD3 in normoxia (**Figure 2A**), this result indicates that the GD3 effect on cell death is not strictly concentration-dependent, suggesting a threshold phenomenon with a critical GD3 level above which stimulates hypoxia-mediated cell death.

Since hypoxia-mediated superoxide anion can be converted mainly by Mn-SOD catalysis into hydrogen peroxide, we next analyzed the levels of both species using HE and DCF staining, respectively. As shown, both species increased under hypoxia in control Hep3B cells transfected with empty vector, being the magnitude of the generation significantly enhanced in Hep3B-GD3 cells upon prolonged exposure to hypoxia (**Figure 2C, D**). Although the time-dependent changes in cell viability and levels of HE and DCF fluorescence suggest that hypoxia exceeds a critical threshold of ROS generation that compromises cell viability in Hep3B-GD3 cells, we cannot discard the generation of other potentially toxic species such as peroxinitrate, which may also contribute to DCF fluorescence.

To verify that the increase in GD3 contributed to the cell death observed in hypoxic conditions, we examined the susceptibility of Hep3B cells to the exogenous administration of GD3 during hypoxia. As observed, GD3 potentiated the death of Hep3B cells following hypoxia incubation, even at low doses at which GD3 had no effect under normoxia (**Figure 2A**). However, higher concentrations of GD3 killed Hep3B independently of the oxygen levels, correlating with higher ROS formation (not shown). Similar findings showing sensitization to hypoxia by exogeneous GD3 exposure were observed in other cancer cell types including human neuroblastoma SH-SY5Y cells (**Figure S5**) or HepG2 cells (not shown), indicating that the sensitizing effect of GD3 is not cell specific. Thus, although other reports indicated that GD3 induces ROS generation [9], [25]–[27], our data indicating that GD3 amplified hypoxia-induced ROS formation and subsequent cell death, do not support a direct role for GD3 in ROS generation in Hep3B-GD3 cells based on the outcome observed in normoxia (**Figure 1D**). Rather, we hypothesized that the potentiation of ROS formation in Hep3B-GD3 cells by hypoxia may have been the result of the interference of NF-κB-mediated antioxidant expression (e.g. Mn-SOD) by GD3, consistent with previous reports showing the ability of gangliosides to prevent NF-κB activation in primary hepatocytes [10] or T cells [12], [25], which is specifically examined next.

### GD3 Synthase Overexpression Prevents c-Src Activation and Subsequent NF-κB Transactivation

**Figure 3:**
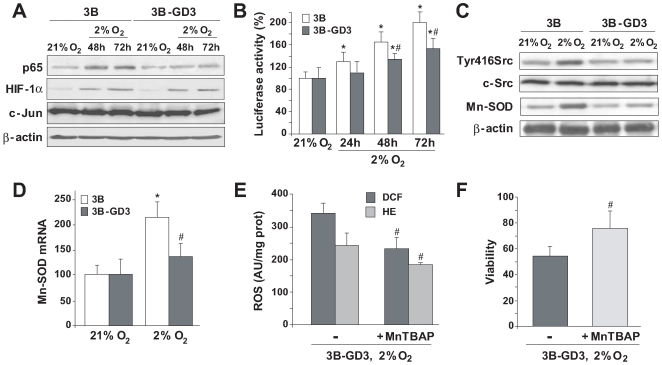
Overexpression of GD3 blocks NF-κB transcriptional activity and downregulates Mn-SOD expression in hypoxic cells. *A.* Representative western blots of p65 and HIF-1α from nuclear extracts of control (3B) and GD3-overexpressing (3B-GD3) cells using c-jun and β-actin as representative nuclear protein and cytosolic marker, respectively (n = 3). *B*, Luciferase activity in 3B and 3B-GD3 cells transfected with NF-κB luciferase reporter construct (n = 4). *C.* Representative immunoblots with c-Src, phospho-Tyr416Src and Mn-SOD antibodies and normalized by β-actin levels. D, Mn-SOD mRNA levels were analyzed by real-time PCR after 72 hours (n = 3). *E,* ROS production, and F, survival of Hep3B-GD3 in the presence or absence of Mn-SOD mimetic, MnTBAP (50 µM) after 72 h hypoxia (n = 4). *p<0.05 vs. Hep3B cells. #p<0.05 vs. hypoxic control.

As suggested in the preceding findings and since ROS production during hypoxia has been shown to participate in NF-κB activation [5], we next examined the effect of GD3 synthase overexpression in p65 nuclear levels. Compared with the nuclear levels of c-Jun as control, p65 nuclear translocation following hypoxia increased in Hep3B cells, and this event was suppressed in Hep3B-GD3 cells (**Figure 3A**). Of note, hypoxia-induced HIF-1α levels were comparable in Hep3B and Hep3B-GD3 cells (**Figure 3A**), and consistent with this outcome, no changes in the mRNA of HIF-dependent genes (pyruvate dehydrogenase kinase 1, PDK1; and vascular endothelial growth factor, VEGF) were detected (data not shown), underscoring the specificity of the inhibition of NF-κB activation by GD3. Moreover, using a reporter luciferase vector with NF-κB consensus sequences in its promoter, we next assessed the changes in transcriptional activity of NF-κB. As observed, luciferase activity increased in Hep3B cells when grown under hypoxia, and this response was significantly attenuated in Hep3B-GD3 cells (**Figure 3B**), thus indicating that GD3 impairs hypoxia-induced NF-κB-mediated gene expression.

Hypoxia has been shown to activate NF-κB through IκB tyrosine phosphorylation via c-Src [5]. Hence, we next checked the status of c-Src in Hep3B-GD3 cells during hypoxia to examine whether this pathway is also susceptible to GD3 inhibition. As seen, the phosphorylation of c-Src in tyrosine 416 induced by hypoxia was observed in Hep3B cells but, unexpectedly, it was abrogated in Hep3B-GD3 cells (**Figure 3C**), suggesting that the interference of GD3 with c-Src stimulation contributed to the suppression of NF-κB activation elicited by hypoxia.

We next focused on the potential consequences of the suppression of the cSrc/NF-κB pathway, specifically analyzing the expression of Mn-SOD, a key antioxidant enzyme that downregulates mitochondrial ROS, which is transcriptionally controlled by NF-κB. As seen, Mn-SOD mRNA levels increased in Hep3B cells following hypoxia, but this response was markedly blunted in Hep3B-GD3 cells (**Figure 3D**). To evaluate if the inhibitory action of GD3 on the cSrc/NF-κB pathway induced by hypoxia was cell type specific or common to other hepatoma cells, HepG2 cells were cultured under hypoxic conditions examining c-Src tyrosine phosphorylation and Mn-SOD expression after GD3 administration. GD3 addition in the last 24 hours of hypoxia exposure blocked the c-Src tyrosine phosphorylation induced by 72 hours of hypoxia (2% O_2_), abrogating the increase of Mn-SOD levels observed in hypoxic cells (**Figure S6**). These results suggest not only that the inhibitory effect of GD3 is not specific for a particular hepatoma cell line, but also that exogenous GD3 antagonizes the c-Src/NF-kB pathway induce by hypoxia, reproducing the effects observed with the stably expression of GD3 synthase in Hep3B-GD3 cells, which minimizes the potential contribution of artifacts during clone selection.

Finally, to evaluate whether Mn-SOD participates in the elimination of the hypoxia-induced ROS production, GD3 synthase-overexpressing cells were incubated with MnTBAP, a porphyrin derivative with antioxidant activities that acts predominantly as a SOD mimetic. Interestingly, MnTBAP drastically reduced the production of superoxide anion and hydrogen peroxide examined by HE and DCF fluorescence, respectively, (**Figure 3E**), and more importantly, MnTBAP significantly protected Hep3B-GD3 cells from hypoxia-induced cell death (**Figure 3F**). The findings showing a decrease in DCF fluorescence by MnTBAP are interesting and somewhat unexpected, as the decrease in superoxide anion by MnTBAP would be anticipated to result in increased DCF fluorescence. However, we speculated that the fate of hydrogen peroxide generated from the scavenging of superoxide anion is governed by the content of mitochondrial GSH. To prove this point, we examined in isolated rat liver mitochondria the levels of superoxide anion and hydrogen peroxide induced by xanthine plus xanthine oxidase (X+XO) with or without mitochondrial GSH depletion upon ethacrinic acid (EA) pretreatment (**Figure S7**). As seen, unlike MitoSox fluorescence, DCF fluorescence increased significantly by MnTBAP only upon mitochondrial GSH depletion by EA, indicating that the status of mitochondrial GSH controls the fate of hydrogen peroxide produced by the scavenging of superoxide anion. In line with these data in isolated rat liver mitochondria, Hep3B and Hep3B-GD3 cells exhibited mitochondrial GSH levels (4–5 nmol/mg protein) which are in the range of those observed in freshly isolated rat liver mitochondria (**Figure S7**), suggesting that the generation of hydrogen peroxide resulting from the scavenging of superoxide anion by MnTBAP is efficiently reduced by an adequate GSH redox system. Moreover, in agreement with previous findings [5], we observed that p65 silencing abrogated Mn-SOD expression during hypoxia resulting in decreased cell viability of Hep3B cells, and that this effect was reversed by MnTBAP. Thus, these findings lend further support for a critical role for Mn-SOD in the susceptibility to hypoxia due to GD3 synthase overexpression, and strongly suggest that GD3 accumulation prevents hypoxia-mediated NF-κB activation by interfering with cSrc/NF-κB survival pathway that mediates MnSOD expression.

### GD3 Synthase Overexpression Reduces *In Vivo* Tumor Growth

**Figure 4:**
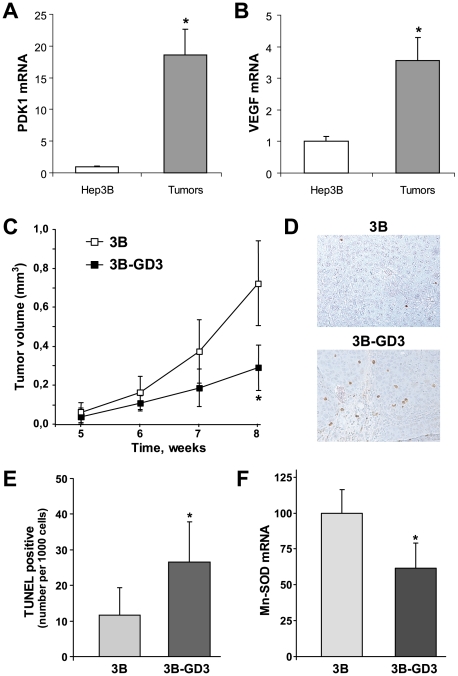
GD3 synthase overexpression decreases subcutaneous tumor growth of Hep3B cells. mRNA levels of PDK1 (*A*) and VEGF (*B*) in Hep3B cells from subcutaneous tumors in nude mice or from cells growing under normal culture conditions with 21% O_2_ (n = 3–4). *p<0.05 vs. normoxic Hep3B cells. *C*, Tumor volume was measured in nude mice injected with Hep3B (empty vector) and Hep3B-GD3 cells (n = 5–6 animals per group). *D, E.* Representative TUNEL-staining in tumor samples from injected mice, and quantification of TUNEL-positive cells in each group as indicated in methods (n = 3-4). *p<0.05 vs. Hep3B-injected mice. *F.* mRNA levels of Mn-SOD from Hep3B and Hep3B-GD3 subcutaneous tumors (n = 4). *p<0.05 vs. Hep3B-injected mice.

A growing body of evidence indicates that HIF-1α contributes to tumor progression and metastasis both in human tumors [28] and xenograph models [29]. In particular, subcutaneous tumors of Hep3B cells in mouse have been demonstrated to grow under hypoxic conditions, and have been used as a model to check potential anticancer HIF-targeting drugs [30]. Moreover, subcutaneous implants of human cervical squamous carcinoma have been validated to grow under hypoxia shown by pimonidazole staining in tumor samples [31]. Nevertheless, we verified in the *in vivo* model that tumor cells were growing in a hypoxic environment by measuring the mRNA levels of well-known HIF-dependent genes such as PDK1 or VEGF, which are controlled by a hypoxia response element (HRE) in their promoters, as well as the HIF-1α expression. As shown, Hep3B cells from subcutaneous tumors displayed a significant increase in the mRNA levels of PDK1 and VEGF compared to Hep3B cultured under normoxic conditions (**Figure 4A, B**), and increased HIF-1α levels (**Figure S8**). Therefore, we next addressed the relevance of the preceding *in vitro* findings by examining their impact in *in vivo* tumor xenografts, in which nude mice were inoculated subcutaneously with Hep3B (with empty vector) or Hep3B-GD3 cells to examine the course of tumor progression and Mn-SOD expression. Tumor volume, determined by a vernier caliper as detailed before [32], increased over time in mice injected subcutaneously with Hep3B cells, while significantly smaller tumors derived from Hep3B-GD3 cells (**Figure 4C**). In addition, we next examined the presence of dying cells in the growing tumors by TUNEL staining. The number of TUNEL positive cells was higher in tumors from Hep3B-GD3-injected cells compared to those of Hep3B cells (**Figure 4D, E**). Although TUNEL staining is indicative of fragmented DNA from dying cells it does not discriminate between necrosis or apoptosis. In addition, there was no difference in active caspase-3-positive cells was found in tumor samples (data not shown). Thus, in agreement with the *in vitro* findings, it appears that necrosis is the predominant form of cell death in the *in vivo* xenograpth model, although at present we cannot rule out the participation of a mitochondrial apoptotic cascade. Moreover, consistent with the suppression of NF-κB activation by hypoxia in the *in vitro* studies, the Mn-SOD mRNA levels were lower in tumor biopsies from mice injected with Hep3B-GD3 cells compared to those from Hep3B xenografts (**Figure 4F**). However, whether this outcome is consequence of the defective c-Src/NF-κB signaling induced by GD3 in the xenograph model, as observed above *in vitro*, remains to be established. All together, these findings indicate a correlation between reduced MnSOD expression and enhanced cell death under hypoxia, which translates in impaired tumor growth in Hep3B-GD3 xenografts.

## Discussion

The present study examines the impact of increased GD3 levels on hypoxia susceptibility *in vitro* and tumor growth *in vivo*. We describe that the overexpression of GD3 synthase alters the pattern of GSLs expression in human hepatocarcinoma cells, with the most significant changes observed being the decrease in GM3 levels and the subsequent increase in GD3 stores. While this shift does not result in changes in cell morphology or cell growth during normoxia, it renders hepatocarcinoma Hep3B cells susceptible to hypoxia-mediated ROS generation and subsequent cell death by suppressing NF-κB-regulated Mn-SOD expression. Although most of this sensitization can be attributable to GD3 overgeneration, another potential contribution to this effect relates to the accompanying downregulation of GM3 levels that is observed in Hep3B-GD3. However, if this were the case one might expect that lower GM3 levels would affect cell viability and growth rate in normoxia, which was not the case; moreover, exogenous GM3 exposure did not reverse hypoxia susceptibility (not shown), thus suggesting that the accompanying GM3 downregulation in Hep3B-GD3 cells played a minor role in our observations. To further support a causal role for GD3 accumulation in hypoxia susceptibility in transfected cells, we observed that exogenous GD3 recapitulated the effects of hypoxia by inducing cell death and by inhibiting the hypoxia-activated c-Src/NF-κB pathway. Although this approach minimizes the potential contribution of artifacts during clone selection, it has been shown that exogenous GD3 can be modified to other sphingolipid species in the endocytic process, even at the membrane location [33]. In particular, plasma membrane-associated sialidases are expressed in the liver, which by removing acid sialic residues may initiate ganglioside catabolism. Consequently, less complex gangliosides and even higher ceramide levels may be detected after exogenous administration of gangliosides. Furthermore, an additional advantage in using the GD3 synthase overexpression approach over the exogenous addition of GD3 is that the former allowed us to examine the *in vivo* relevance of intracellular GD3 accumulation in tumor growth in xenografts.

**Figure 5:**
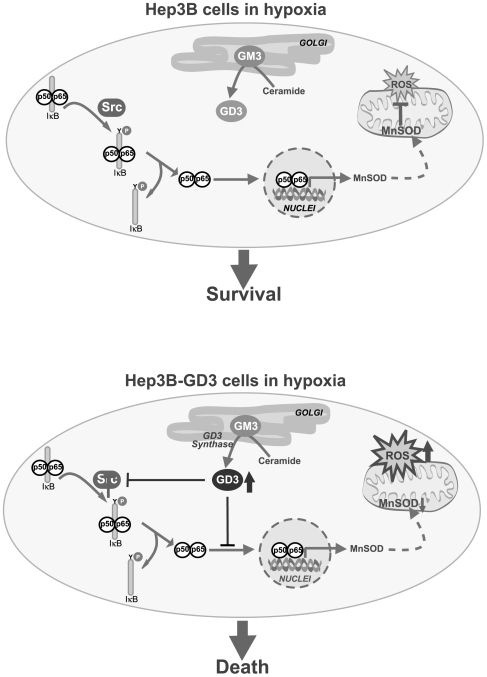
Schematic diagram depicting the mechanism proposed to be involved in the susceptibility of Hep3B cells to hypoxia upon GD3 synthase overexpression. In Hep3B cells, NF-κB activation via cSrc plays a key role in the resistance to hypoxia, in part, by inducing Mn-SOD expression. However, in Hep3B-GD3 cells the overgeneration of GD3 blocks the cSrc/NF-κB pathway potentiating the mitochondrial ROS production during hypoxia and contributing to hypoxia-mediated cell death.

In addition to the interference of NF-κB activation by GD3, which has been observed in the past in various cell types [11], [12], [16], [25], a salient feature of the present study is the ability of GD3 to disable the activation of c-Src induced by hypoxia, in agreement with findings in epithelial cell adhesion and spreading, in which GD3 blocked c-Src activation [34]. Due to the crucial role of c-Src in NF-κB activation during hypoxia [5], the block of c-Src activation emerges as a novel mechanism whereby GD3 inactivates NF-κB, although a direct effect on DNA-competent κB members by GD3 shown previously [11], [16] could contribute to the final outcome of impaired κB-mediated gene expression and subsequent attenuation of survival pathways (**Figure 5**). As the nuclear localization sequence (NLS) of κB members is essential for their nuclear translocation, we can postulate that GD3 may interfere with this signal preventing their movement to the nuclei. Whether GD3 directly interferes with a cluster of positive aminoacids, part of the NLS involved in the nuclear translocation of κB members, deserves further investigation. The mechanism whereby GD3 prevents hypoxia-induced c-Src activation is currently unknown. However, given the role of ROS in hypoxia-mediated c-Src activation [5], our findings suggest the involvement of a direct modulatory effect of GD3 on c-Src independently of ROS generation. In this regard, it has been previously found that gangliosides inhibit tyrosine phosphorylation [35], particularly GD3, which has been shown to regulate the activation of Lyn, a member of the Src family, by their interaction in caveolae-like domains [36]. More recently it has been proposed that GD3 participates in a multimolecular complex, composed of sphingolipid-enriched membrane domains enriched in Src family protein tyrosine kinases [37], and that alterations in the composition of these domains affects c-Src activation. Whether a similar mechanism is involved in our observations with Hep3B-GD3 cells deserves further investigation.

Previous studies have shown that GD3 interacts with mitochondria, eliciting a burst of ROS generation [9], [10], [23], [25]. Interestingly, although GD3 trafficked to intracellular compartments in Hep3B-GD3 cells including mitochondria, the fact that no significant ROS generation is observed during normoxia suggests that that mitochondrial localization of GD3 upon GD3 synthase overexpression may seem insufficient to stimulate ROS production in Hep3B-GD3. Our results, however, suggest that mitochondria constitute the main source for the observed ROS generation during hypoxia, consistent with previous findings [4], [5], [24] and with the protection observed with MnTBAP, which in the presence of GD3 results in the sensitization of Hep3B-GD3 cells. Thus, the combination of mitochondrial ROS generation (induced by hypoxia) and the suppression of c-Src-mediated NF-κB activation (by GD3) synergize to the sensitization of Hep3B-GD3 to hypoxia (**Figure 5**). A caveat in the study relates to the uncertainty in the mechanism of action of the SOD mimetic MnTBAP. While its predominant function is as a SOD mimetic scavenging superoxide anion, other reports indicated its potential to scavenge peroxinitrate as well [38]. Regardless of its true mechanism of action, the benefit of MnTBAP has been clearly documented in Sod2^tm1Cje^ null mice, in which MnTBAP treatment prevented systemic pathology (dilated cardiomyopathy, hepatic lipid accumulation), dramatically extending lifespan [39]. These data indicate that this antioxidant is able to functionally replace Mn-SOD deficiency, suggesting that a predominant mechanism in the protective effects of MnTBAP is via superoxide anion elimination.

Our findings may be of therapeutic significance *in vivo* as illustrated in hepatocarcinoma xenografts, in which tumor growth and Mn-SOD expression were lower in Hep3B-GD3-derived tumors. Whether or not it may be of potential relevance in the clinical setting remains to be established, and experiments analyzing the ganglioside profile in primary human liver tumors in comparison with healthy liver biopsies may help to clarify this important issue. Interestingly, Ruckhäberle et al observed a worst prognosis for breast cancer patients exhibiting tumors showing low expression of GD3 synthase [40].

In conclusion, GD3 synthase overexpression in a human HCC cell line catalyzes the conversion of endogenous GM3 stores into GD3, which distributes to different cell sites. While GD3 increase does not perturb cell growth/death during normoxia, it sensitizes Hep3B cells to hypoxia-induced ROS generation, due to the ability of GD3 to disable the cSrc/NF-κB pathway by an as yet uncharacterized mechanism that translates in lower Mn-SOD expression. Based on previous findings showing mitochondria as the source of ROS during hypoxia [4], [5], [24], we speculate that mitochondria constitute the site of ROS overproduction in Hep3B. In line with this possibility, MnTBAP, which has been shown to be able to replace Mn-SOD deficiency *in vivo*, protected Hep3B-GD3 cells from hypoxia-induced ROS overproduction. Taken together, these findings mirrored the outcome observed in Hep3B-GD3 xenografts, suggesting the potential relevance of GD3 synthase overexpression in *in vivo* tumor growth and identify GD3 as a potential relevant therapeutic agent to turn hypoxia into a cancer-threatening milieu.

## Materials and Methods

### Cell Culture and Incubation

Human hepatocarcinoma Hep3B and HepG2 cells as well as the human neuroblastoma SH-SY5Y cells were obtained from the ECACC and cultured in a humidified incubator (RS Biotech) at 37°C and 5% CO_2_ either at 21% O_2_ (normoxia) or 2% O_2_ (hypoxia) in sealed flasks for 24–72 hours as previously described [5], [40]. Hypoxia was achieved in the incubator by displacing O_2_ with injected N_2_ using an Active Oxygen Control that allow to maintain the O_2_ environment between 1% and 19% with a 0.1% accuracy. In some cases, Hep3B and Hep3B-GD3 cells were incubated in the presence of MnTBAP (50 µM), pan-caspase inhibitor qVD-OPH (20 µM) or GD3 ganglioside (0–50 µM, stock solution of 10 mM in ethanol) for the last 24 h of hypoxia to examine their role in hypoxia susceptibility. The addition of solvent alone used to dissolve GD3 to Hep3B did not affect cell viability in normoxia or hypoxia (not shown).

### Glycosphingolipids TLCs and GD3 Synthase Enzyme Activity Assay

Cells (2–3×10^6^) were harvested by scraping and resuspended in 50 µl of 10 mM sodium cacodylate buffer (pH 7.0). Samples were subjected to lysis with an equal volume of 0.6% Triton CF-54 in 50 mM sodium cacodylate buffer and 20 mM MgCl_2_ (assay buffer) during 20 min at 4°C and centrifuged for 10 min at 14000 g. Supernatants (50 µl/activity assay) were supplemented with 15 µl of 2 mM GM3, 15 µl of 70 µM CMP-[^14^C] sialic acid (0.3 µCi) and 20 µl of assay buffer and incubated for 3 h at 37°C. Blanks assays were performed with the heat-inactivated enzyme. Radiolabelled glycolipids were separated by Sephadex G-50 gel filtration and divided into two equivalents portions: the first one was dried and counted in a scintillation spectrometer and the remaining portion was subjected to TLC. In addition, for the measurement of glycosphingolipid intracellular levels, cells were isotopically labeled with ^3^H-galactose 1 µCi/ml for 24 hours before lipid extraction and TLC analyses. Samples were run in parallel with commercial standards (Matreya) and TLC plates developed after spraying with 0.1% orcinol dissolved in 5% H_2_SO_4_, and heated at 120°C for approximately 10 minutes.

### Plasmid Construction and Cell Transfection

Full-length cDNA of GD3 synthase (Accession Number: NM_003034) was obtained from the human glioblastoma cell line U373 with the SuperScript preamplification Kit (Invitrogen) by PCR using the primers: forward 5′-CACCATGAGCCCCTGCGGGCGG-3′ and reverse 5′- GGAAGTGGGCTGGAGTGAGGTATC-3′. The PCR product (1.07 kb) was cloned into pcDNA 3.1D/v5-His-TOPO with the directional TOPO expression kit (Invitrogen) and verified by DNA sequencing. Hep3B were transfected using Lipofectamine 2000 (Invitrogen) with the full-length construct (Hep3B-GD3) or with the empty vector (Hep3B). 23 different colonies overeexpressing GD3 synthase were selected based on their resistance to 1 mg/ml G418 (Roche) during 4 weeks, while a pool of Hep3B cells trasfected with the empty vector were used as control in all the experiments. The GD3 synthase-expressing colony with higher enzymatic activity was chosen, although other colonies with similar activity were also tested, obtaining similar results in terms of hypoxia susceptibility and c-Src/NF-κB regulation.

### Measurement of ROS and Cytotoxicity

Hydroethidine (HE) (ex 495/em 610) and 2′-7′-dihydro dichlorofluorescein diacetate (DCF) (ex 495/em 525) were from Molecular Probes. Fluorescence was determined at 37°C and data normalized with values from normoxic untreated controls [5]. Cytotoxicity was determined by MTT assay or through the leakage of lactate dehydrogenase (LDH) into the medium. In addition, cell viability was measured by exclusion of propidium iodide (15 µg/ml; Sigma) after flow citometry analysis of non-permeabilized cells.

### Cell-Cycle Analysis

After 48–72 hours of hypoxic treatment, cells were fixed and permeabilized in 100% ethanol, washed with PBS and stained with propidium iodide (15 µg/ml) in the presence of 0.3 mg/ml Ribonuclease A (Sigma). Cell-cycle distribution was assayed using a BD FACSCalibur flow cytometer (Becton Dickinson).

### Real-Time RT-PCR

Briefly, 20 ng of total RNA, 600 µmol/l of primers, and 12.5 µl of 2X Reaction Mix (Sensimix One-Step, Quantance) were incubated in 25 µl at 50°C for 10 min and 95°C for 5 min, followed by 45 cycles of 95°C for 10 s, 56°C for 30 s, and 72°C for 30 s. Each reaction was run in duplicate and the threshold (CT) values for Mn-SOD mRNA (BC012423), PDK1 (NM_002610.3) or VEGF (NM_001025366.1|) were subtracted from that of β-actin mRNA (NM_001101), averaged and converted from log-linear to linear term. The primer sequences used were:

β-actin forward 5′-TTGCCGACAGGATGCAGAA-3′,

β-actin reverse 5′-GCCGATCCACACGGAGTACT-3′,

Mn-SOD forward 5′- CCACCACCATTGAACTTCAG-3′,

Mn-SOD reverse 5′-GGCTGAGGTTTGTCCAGAAA-3′.

PDK1 forward 5′-GAAGCAGTTCCTGGACTTCG-3′,

PDK1 reverse 5′-ACCAATTGAACGGATGGTGT-3′;

VEGF forward 5′-CTACCTCCACCATGCCAAGTG-3′,

VEGF reverse 5′-TGCGCTGATAGACATCCATGA-3′.

### Cell Transfection with Reporter Construct and siRNA Silencing

Briefly, 5×10^5^ were plated onto a 24 wells culture plates and transiently transfected using Lipofectamine™ 2000 (Invitrogen, Carlsbad, CA) with the reporter plasmid pNF-κB-Luc and phRL-TK for normalization. Cells were harvested 48–72 h after transfection, and the Firefly and Renilla luciferase activities were measured with the dual luciferase assay system according to the manufacturer's instructions (Promega). GD3 synthase RNA silencing was achieved in Hep3B cells with target- specific siRNA duplexes (Santa Cruz) using Lipofectamine™ 2000 as transfecting agent and its related scramble siRNA as a control. Protein and mRNA levels from siRNA-transfected cells were analyzed after 48 hours of transfection, while cell growth was determined during 72 hours.

### Immunofluorescence

Cells grown in coverslips were rinsed with PBS, fixed and permeabilized in acetone at -20°C for 10 min. After incubation with PBS containing 5% BSA for 1 hour, monoclonal anti-GD3 antibody R24 (Calbiochem, 1∶150 in PBS-BSA) was added at 4°C overnight. In some instances, cells were labeled in parallel with anti-Mn-SOD antibody to examine the colocalization of GD3 with mitochondria. Coverslips were washed with PBS, incubated with Alexa Fluor 488 anti-mouse (Molecular Probes, 1∶500, 1 h, RT), and mounted in DakoCytomation fluorescent mounting medium. Confocal images were collected using a Leica SP2 laser scanning confocal microscope equipped with UV excitation, an argon laser, a 633/1.32 OIL PH 3 CS objective and a confocal pinhole set at 1 Airy unit. All the confocal images shown were single optical sections.

### Western Blotting

Blots were incubated with anti-P-Tyr 416 Src antibody (Cell Signaling), anti-c-Src, anti-MnSOD2, anti-GD3 Synthase antibodies (Santa Cruz) and anti-β-actin antibody (Sigma). Nuclear extracts, obtained as described [5], were immunoblotted with anti-p65, c-jun antibodies (Santa Cruz) and anti-HIF-1α (Novus Biologicals).

### Hepatocarcinoma Xenograft Model

All procedures were performed according to protocols approved by the IDIBAPS Ethical Committee.

Six-week-old male BALB/c athymic (nu/nu) nude mice, kept under pathogen-free conditions and given free access to food and sterilized water, were s.c. injected with control Hep3B or GD3-expressing (Hep3B-GD3) cells (2.5×10^6^ cells in 200 µL of PBS) examining tumor growth with a vernier caliper, as described previously [27]. After 8 weeks, samples were taken for mRNA and TUNEL analysis using the In Situ Cell Death Detection kit (Roche) as previously described [42]. Quantification was performed in 10 random-selected fields per tumor with at least 200 cells/field each. mRNA from Hep3B cells growing subcutaneously during 8 weeks in nude nice, and from Hep3B cells cultured under normal conditions (21% O_2_, 5% CO_2_) were extracted simultaneously and the mRNA expression of HIF-dependent genes (PDK1 and VEGF) analyzed to verify the existence of hypoxic conditions in our tumor samples.

### Rat Liver Mitochondria Isolation, GSH Depletion and ROS Generation

In order to address the role of GSH in the generation of hydrogen peroxide from the scavenging of superoxide anion by MnTBAP, rat liver mitochondria, isolated as previously described [9], [32], were incubated with xanthine plus xanthine oxidase (0.1 mM plus 20 U/ml), as detailed previously [43], with or without ethacrinic acid (0.5 mM) to deplete GSH levels. Mitochondria were then incubated with or without MnTBAP and the generation of superoxide anion and hydrogen peroxide were determined [43].

### Statistics

Results were expressed as mean ± SD. Statistical significance of the mean values was established by the two-tailed distribution Student's t test.

## Supporting Information

Figure S1 (1.37 MB PDF)

Figure S2 (0.14 MB PDF)

Figure S3 (0.14 MB PDF)

Figure S4 (0.21 MB PDF)

Figure S5 (0.16 MB PDF)

Figure S6 (1.31 MB PDF)

Figure S7 (0.16 MB PDF)

Figure S8 (0.70 MB PDF)
