# Supplementary material for: GD3 Synthase Overexpression Sensitizes Hepatocarcinoma Cells to Hypoxia and Reduces Tumor Growth by Suppressing the cSrc/NF-κB Survival Pathway
Source: PLoS One. 2009 Nov 26;4(11):e8059. doi: 10.1371/journal.pone.0008059 (PMC2777380; doi:10.1371/journal.pone.0008059)
Supplement: Figure S1 — (1.37 MB PDF) [file pone.0008059.s001.pdf]

Supplemental Figure 1

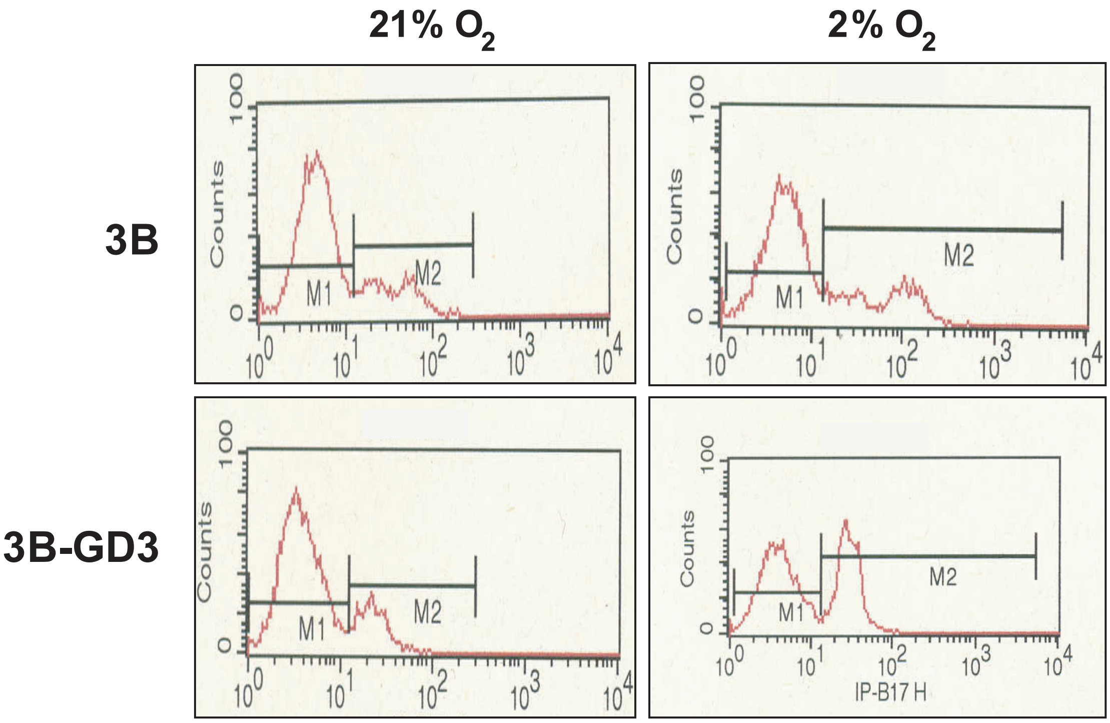

Cell death was evaluated by flow cytometry after staining with propidium iodide of non-permeabilized Hep3B and Hep3B-GD3 cells exposed during 72 hours to normoxia (21% O_2) or hypoxia (2% O_2). Only GD3 Synthase overexpressing HEP3B cells after hypoxia presented an increased M2 population with higher fluorescence (propidium iodide positive).
