# Supplementary material for: GD3 Synthase Overexpression Sensitizes Hepatocarcinoma Cells to Hypoxia and Reduces Tumor Growth by Suppressing the cSrc/NF-κB Survival Pathway
Source: PLoS One. 2009 Nov 26;4(11):e8059. doi: 10.1371/journal.pone.0008059 (PMC2777380; doi:10.1371/journal.pone.0008059)
Supplement: Figure S2 — (0.14 MB PDF) [file pone.0008059.s002.pdf]

Supplemental Figure 2

FACS (PI Staining)		Cell Cycle Distribution (%)		
Cells	Treatment	G0/G1	S	G2/M
3B	21% O_2	60 ± 5.1	15 ± 4.8	20 ± 3.4
3B-GD3	21% O_2	63 ± 6.4	20 ± 3.3	15 ± 2.0
3B	48h, 2% O_2	65 ± 4.8	15 ± 2.8	15 ± 2.4
3B-GD3	48h, 2% O_2	70 ± 3.7	16 ± 3.3	10 ± 3.5
3B	72h, 2% O_2	64 ± 5.2	17 ± 4.1	14 ± 2.8
3B-GD3	72h, 2% O_2	69 ± 6.2	15 ± 3.9	13 ± 1.9

Cell cycle distribution was evaluated by flow cytometry after staining with propidium iodide of Hep3B and Hep3B-GD3 cells exposed during diferent times to normoxia (21% O_2) or hypoxia (2% O_2). Cells were fixed and permeabilized in 100% ethanol, washed with PBS and stained with propidium iodide (15 mg/ml) in the presence of 0.3 mg/ml Ribonuclease A.
