# Supplementary material for: GD3 Synthase Overexpression Sensitizes Hepatocarcinoma Cells to Hypoxia and Reduces Tumor Growth by Suppressing the cSrc/NF-κB Survival Pathway
Source: PLoS One. 2009 Nov 26;4(11):e8059. doi: 10.1371/journal.pone.0008059 (PMC2777380; doi:10.1371/journal.pone.0008059)
Supplement: Figure S3 — (0.14 MB PDF) [file pone.0008059.s003.pdf]

Supplemental Figure 3

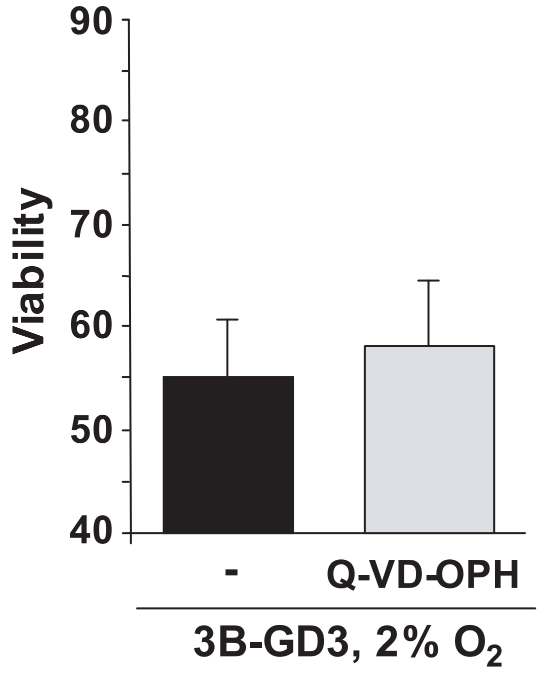

Cell viability of Hep3B-GD3 cells under hypoxia for 3 days treated with or without the pan-caspase inhibitor Q-VD-OPH (20 μM). (n=2).
