# Supplementary material for: GD3 Synthase Overexpression Sensitizes Hepatocarcinoma Cells to Hypoxia and Reduces Tumor Growth by Suppressing the cSrc/NF-κB Survival Pathway
Source: PLoS One. 2009 Nov 26;4(11):e8059. doi: 10.1371/journal.pone.0008059 (PMC2777380; doi:10.1371/journal.pone.0008059)
Supplement: Figure S4 — (0.21 MB PDF) [file pone.0008059.s004.pdf]

Supplemental Figure 4

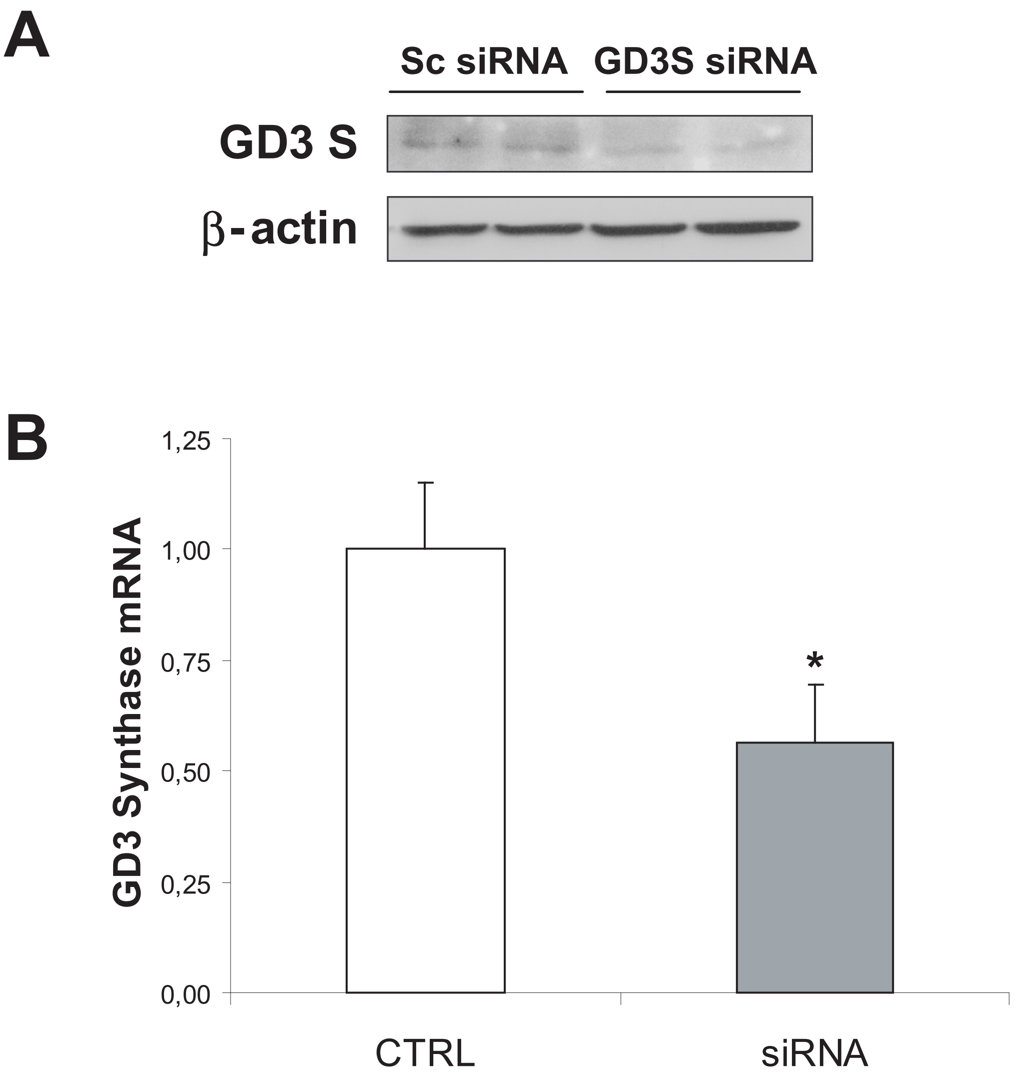

Western blot (panel A) and mRNA levels (panel B) of GD3 Synthase in Hep3B cells transfected with scramble (Sc) or specific GD3 Synthase (GD3S) siRNAs and measured 48 hours later (n=3). *p<0.05 vs. CTRL Hep3B cells.
