# Supplementary material for: GD3 Synthase Overexpression Sensitizes Hepatocarcinoma Cells to Hypoxia and Reduces Tumor Growth by Suppressing the cSrc/NF-κB Survival Pathway
Source: PLoS One. 2009 Nov 26;4(11):e8059. doi: 10.1371/journal.pone.0008059 (PMC2777380; doi:10.1371/journal.pone.0008059)
Supplement: Figure S5 — (0.16 MB PDF) [file pone.0008059.s005.pdf]

Supplemental Figure 5

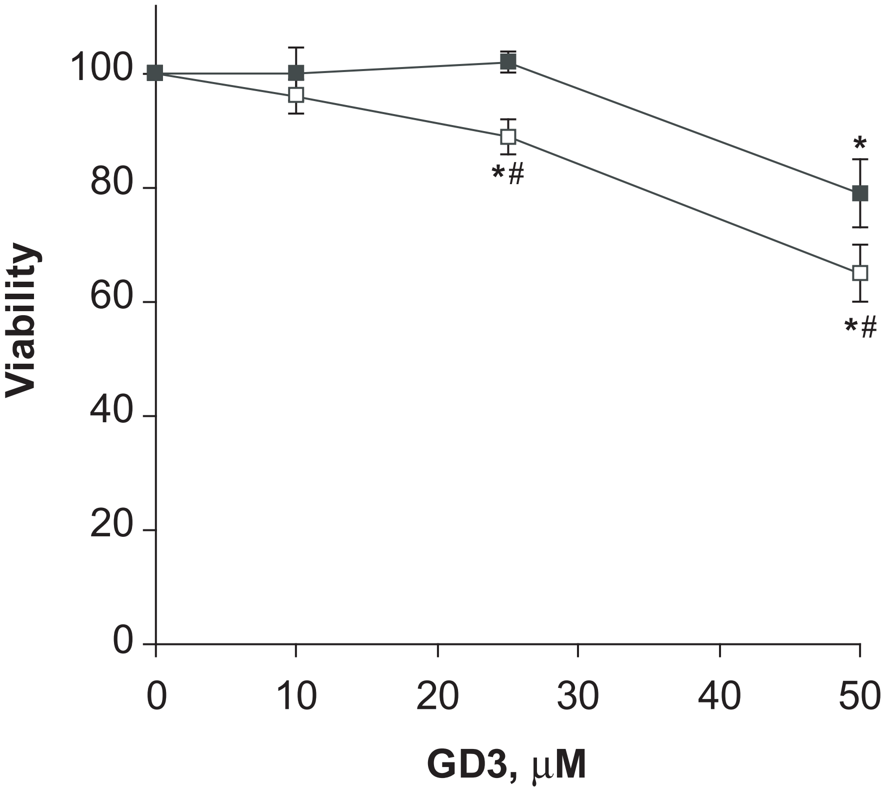

Cell viability of SH-SY5Y cells growing under normoxia or hypoxia ($2\%\ O_2$) for 72 h, and treated with increasing concentrations of GD3 for the last 24 hours (n=2). *$p<0.05$ vs. untreated SH-SY5Y cells. $^{\#}p<0.05$ vs. GD3-treated normoxic SH-SY5Y cells.
