# Supplementary material for: GD3 Synthase Overexpression Sensitizes Hepatocarcinoma Cells to Hypoxia and Reduces Tumor Growth by Suppressing the cSrc/NF-κB Survival Pathway
Source: PLoS One. 2009 Nov 26;4(11):e8059. doi: 10.1371/journal.pone.0008059 (PMC2777380; doi:10.1371/journal.pone.0008059)
Supplement: Figure S6 — (1.31 MB PDF) [file pone.0008059.s006.pdf]

# Supplemental Figure 6

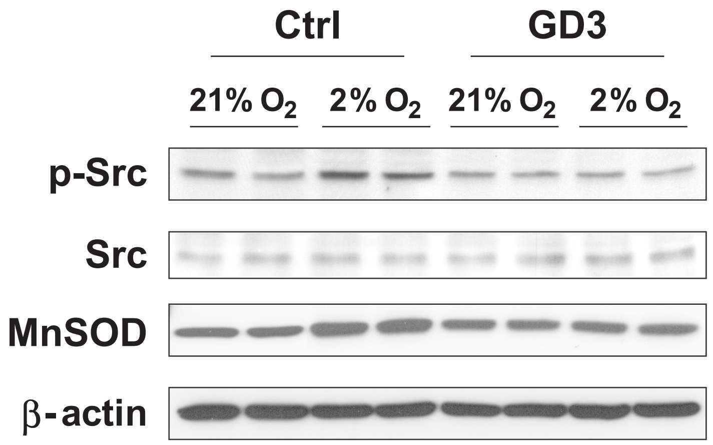


Representative western blot performed with cell extracts from HepG2 cells exposed during 72 hours to normoxia (21% $O_2$) or hypoxia (2% $O_2$) that were treated for the last 24 hours with GD3 or vehicle (ethanol, 0.5%) (n=2).
