# Supplementary material for: GD3 Synthase Overexpression Sensitizes Hepatocarcinoma Cells to Hypoxia and Reduces Tumor Growth by Suppressing the cSrc/NF-κB Survival Pathway
Source: PLoS One. 2009 Nov 26;4(11):e8059. doi: 10.1371/journal.pone.0008059 (PMC2777380; doi:10.1371/journal.pone.0008059)
Supplement: Figure S7 — (0.16 MB PDF) [file pone.0008059.s007.pdf]

Supplemental Figure 7

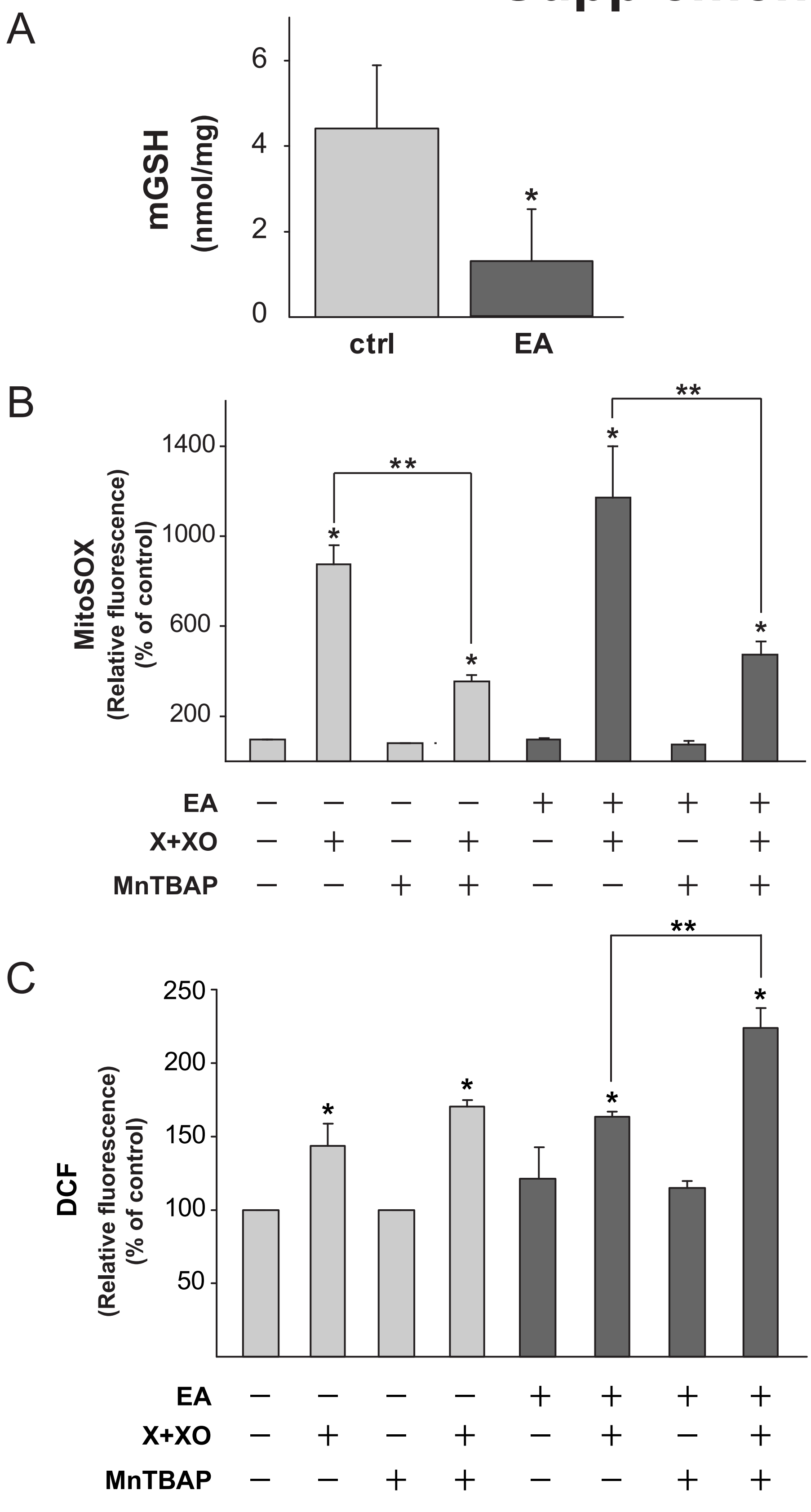

Increase in H_2O_2, but not superoxide, after X/XO exposure concomitant with MnTBAP incubation in rat liver mitochondria after GSH depletion. **A**, mitochondrial GSH was determined in liver mitochondria treated with ethacrinic acid (EA, 250 µM) or vehicle (PBS). **B**, superoxide formation was assessed by MitoSOX fluorescence analysis and reported as relative fluorescence in percent of control mitochondria. **C**, DCF fluorescence was used as an indicator for mainly mitochondrial hydrogen peroxide production, reported as relative fluorescence in percent of control. Data are expressed as means ± SD (n=3-4). *, $p < 0.05$ compared to control; **, $p < 0.05$ compared to respective control without MnTBAP incubation.
