# Supplementary material for: GD3 Synthase Overexpression Sensitizes Hepatocarcinoma Cells to Hypoxia and Reduces Tumor Growth by Suppressing the cSrc/NF-κB Survival Pathway
Source: PLoS One. 2009 Nov 26;4(11):e8059. doi: 10.1371/journal.pone.0008059 (PMC2777380; doi:10.1371/journal.pone.0008059)
Supplement: Figure S8 — (0.70 MB PDF) [file pone.0008059.s008.pdf]

Supplemental Figure 8

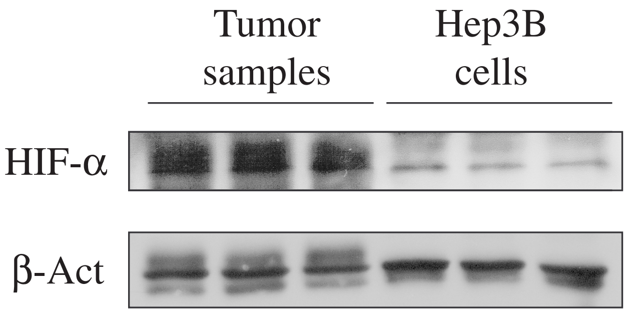

Western blot to evaluate HIF-α protein levels in tumor samples from mice subcutaneously injected with Hep3B cells compared with the expression of Hep3B cells growing under normoxic conditions (21% O_2). Protein levels of β-Act are used as a loading control.
